# Visuospatial Tasks Affect Locomotor Control More than Nonspatial Tasks in Older People

**DOI:** 10.1371/journal.pone.0109802

**Published:** 2014-10-06

**Authors:** Jasmine C. Menant, Daina L. Sturnieks, Matthew A. D. Brodie, Stuart T. Smith, Stephen R. Lord

**Affiliations:** 1 Falls and Balance Research Group, Neuroscience Research Australia, Sydney, Australia; 2 Faculty of Medicine, University of New South Wales, Sydney, Australia; 3 Faculty of Health Science, University of Tasmania, Launceston, Australia; University Children's Hospital Tuebingen, Germany

## Abstract

**Background:**

Previous research has shown that visuospatial processing requiring working memory is particularly important for balance control during standing and stepping, and that limited spatial encoding contributes to increased interference in postural control dual tasks. However, visuospatial involvement during locomotion has not been directly determined. This study examined the effects of a visuospatial cognitive task versus a nonspatial cognitive task on gait speed, smoothness and variability in older people, while controlling for task difficulty.

**Methods:**

Thirty-six people aged ≥75 years performed three walking trials along a 20 m walkway under the following conditions: (i) an easy nonspatial task; (ii) a difficult nonspatial task; (iii) an easy visuospatial task; and (iv) a difficult visuospatial task. Gait parameters were computed from a tri-axial accelerometer attached to the sacrum. The cognitive task response times and percentage of correct answers during walking and seated trials were also computed.

**Results:**

No significant differences in either cognitive task type error rates or response times were evident in the seated conditions, indicating equivalent task difficulty. In the walking trials, participants responded faster to the visuospatial tasks than the nonspatial tasks but at the cost of making significantly more cognitive task errors. Participants also walked slower, took shorter steps, had greater step time variability and less smooth pelvis accelerations when concurrently performing the visuospatial tasks compared with the nonspatial tasks and when performing the difficult compared with the easy cognitive tasks.

**Conclusions:**

Compared with nonspatial cognitive tasks, visuospatial cognitive tasks led to a slower, more variable and less smooth gait pattern. These findings suggest that visuospatial processing might share common networks with locomotor control, further supporting the hypothesis that gait changes during dual task paradigms are not simply due to limited attentional resources but to competition for common networks for spatial information encoding.

## Introduction

Dual task studies requiring individuals to simultaneously perform cognitive and balance tasks have shown that performance in either or both tasks is compromised [1], [2], and that this interference is more marked in those with reduced sensorimotor and/or cognitive functioning due to age and disease [1], [2]. These findings suggest that balance control requires higher-level cognitive functioning to selectively process information (attention) and actively maintain and manipulate it (working memory) [3].

Two models have been proposed to explain the interference between balance and secondary cognitive tasks [1]: the general capacity sharing/limited attentional resources model; and the more specific bottleneck model whereby both the primary balance task and the secondary cognitive task concurrently require similar networks. Concurrent motor timing tasks provide an example of bottleneck processing; e.g. performance of an eye blink classical conditioning task is disrupted by concurrent finger tapping, another task requiring cerebellar processing, but not by a recognition task [4]. Some previous studies have contrasted the effects of visuospatial (VS) and nonspatial (NS) cognitive tasks on balance as a means of determining which of the two models is more apt in this context. Secondary VS cognitive tasks have been shown to reduce balance control during standing and stepping more than NS cognitive tasks in several [4]–[12], but not all [13] previous studies. Potential factors that might have contributed to these conflicting findings include the set order of administration of the cognitive tasks [8], [9], unequal secondary task difficulty [7], [9], [10], [13] and the inconsistent requirement of articulatory responses across trials [8], [9] (a motor task that can detrimentally affect postural control) [14].

There is also some evidence that impaired VS processing is associated with gait instability and falls. Increased dual task cost of walking while completing a VS decision reaction time task was more strongly associated with recurrent falls than a simple reaction-time task to an auditory stimulus in 377 older people [15]. In another large sample of healthy older adults, poor performance in a VS pen and paper test was significantly and independently associated with increased double-support phase variability during gait [16], as well as with increased risk of multiple falls [17]. In Parkinson’s disease patients with mild cognitive impairment, impaired VS processing has been shown to be significantly associated with gait instability [18]. VS processing impairments have also been associated with stepping arhythmicity and asymmetry in people with Parkinson’s disease [19], and in contrast to tests of executive functioning, differentiate between freezers and non-freezers [19]. However, only one of the above studies has directly contrasted the effects of VS versus NS secondary tasks on gait parameters [10], and it is possible the differential effects reported may not be attributable to differences in the task type, as the difficulty of the secondary tasks were not examined or controlled for.

The aim of the present study was to compare the effects of VS and NS cognitive tasks on temporo-spatial gait parameters, smoothness and variability in older people while controlling for task difficulty. We hypothesized that if the control of walking is dependent upon VS working memory processing, walking while performing a cognitive task that requires VS processing, will disrupt one or both tasks (i.e. the bottleneck model) more so than a NS arithmetic task. While both VS and NS tasks call upon a central executive, we used the VS star-movement task [11] to involve the VS sketch pad of working memory (VS task) and a series of single digit additions and subtractions to place demands on the phonological store of working memory (NS task). Determining any differential effects of VS and NS dual tasks may provide insight into cognitive processing pathways utilized in the control of locomotion. Further, given that falls in older people frequently occur while walking [20], understanding the relative importance of secondary cognitive task types in influencing locomotor control has implications for fall risk assessments.

## Methods

### Ethics Statement

The study was conducted in accordance with the Declaration of Helsinki and was approved by the Human Research Ethics Committee at the University of New South Wales. Written informed consent was obtained from all participants prior to commencing the study.

### Participants

Thirty-six older people (18 males; mean (SD) age: 81.4 (3.5) years) volunteered to participate in this study. All lived independently in the community and were part of a larger prospective study of falls, for which they were recruited via random selection from the electoral roll (Sydney Memory & Ageing Study) [21]. Participants had a mean Mini-Mental State Examination (MMSE) [22] score of 28.9 (SD = 1.4), were independent in activities of daily living and were able to walk 400 m without assistance. The participants had on average 14.0 (3.6) years of education. Four participants had fallen two times or more in the 12 months preceding the assessment. The exclusion criteria were medical or psychological conditions that may have prevented participants from completing assessments, i.e. a previous diagnosis of dementia or developmental disability, psychotic symptoms, Parkinson’s disease, multiple sclerosis, motor neuron disease and central nervous system inflammation.

### Experimental set-up

Participants completed a series of three walking trials at self-selected speed along a 20-m long walkway under four conditions presented in a block-randomised order: 1) with an easy arithmetic task (NS-easy); 2) with a difficult arithmetic task (NS-diff); 3) with an easy VS task (VS-easy); and 4) with a difficult VS task (VS-diff). Participants also performed three walking trials with no cognitive task for reference purposes. The cognitive task instructions were delivered through headphones; the instruction durations were as follows: NS-easy 4.7 s, NS-diff 6.3 s, VS-easy 7.4 s, VS-diff 8.5 s. These differences are largely due to a longer (non-informative) lead in time in the VS tasks (i-e: “the star starts in box …”), which took 2.8 s, and no lead in time ahead of the first piece of information in the NS tasks, that being the first number of the sum (i-e: “three plus four plus one”). Instructions were given continuously throughout the walking trial; that is, a new instruction was delivered within a second of the participant answering the previous question. This procedure was identical for all dual task trials and ensured that regardless of their walking speed, participants were cognitively engaged throughout the trial. Participants were instructed to “keep walking normally” and to “give the answers as quickly as possible”. Participants also completed three seated 30 s trials for each cognitive task condition that were randomly presented amongst the blocks of walking trials.

### Cognitive tasks conditions

#### Visuospatial tasks

The VS star movement task has been previously described [11]. In brief, the VS-easy task involved participants envisaging three boxes side by side labelled A, B and C. Participants were shown the empty boxes on a visual display during the explanation of the protocol and were asked to visualise a star located in one of the boxes making three movements. They were then allowed sufficient practice with and then without the visual display until they demonstrated that they understood the test requirements and scored three consecutive correct responses. Pre-recorded instructions delivered the random starting position and the direction of the three movements, i.e. left or right. In the VS-diff task, participants were asked to visualise the star moving among four boxes arranged in a square. The pre-recorded instructions delivered the random starting position and four movements of the star, which comprised up, down, left, right and diagonal moves. As with the VS-easy task, participants practiced the task initially with and then without a visual aid.

#### Nonspatial tasks

The NS-easy task required participants to sum three single digit numerals. The NS-diff task involved a calculation of four single digit numerals comprising both additions and substractions (but with a running total that was always >0). Particants practiced both easy and difficult NS tasks before commencing the data collection trials.

As the main purpose of our study was to compare the effects of different types of secondary cognitive tasks (VS versus NS) on gait parameters, the secondary tasks selected required equivalent verbal responses of mostly one syllable (eg: “nine”, “ten”, “a”, “b”, etc). Thus any confounding effect of speech on gait would be consistent across all dual task conditions.

### Data collection

Pelvis accelerations were recorded by one tri-axial accelerometer (Opal, APDM Inc, Portland, OR, USA; sampling frequency 128 Hz) attached at the level of the sacrum as previously described [23]. Acceleration data collection and processing were performed in custom-written software (MATLAB R2011, Mathworks, Natick, MA, USA).

### Data analysis

#### Gait parameters

The accelerometer data were analysed for the middle 15 m of the 20 m walkway (i.e. constant walking velocity). Heel strike was identified from the characteristic peak anterior acceleration, as reported previously [24] and used to calculate step times between successive heel-strikes and step time variability.

The following variables were computed:

1. Gait speed (m.s^−1^), step length (cm) and cadence (steps.s^−1^).
2. Step time variability (coefficient of variation of step time = (standard deviation of mean step time/mean step time) ×100; %).
3. Vertical, anterior-posterior (AP) and medio-lateral (ML) pelvis harmonic ratios [23]. Harmonics were extracted from the pelvis acceleration data through finite Fourier series. Harmonic ratios were calculated by dividing the sum of the amplitudes of the first ten even harmonics by the sum of the amplitudes of the first ten odd harmonics over one stride for each plane, AP, ML and vertical. Harmonic ratios provide a measure of walking stability or smoothness, as they are based on the assumption that upper body oscillations are repetitive during normal walking [25]; higher harmonic ratios indicate increased stability.

#### Cognitive task performance

Task difficulty was assumed to be reflected in task performance measures, i.e. the time taken to respond and the accuracy of responses to the cognitive tasks. Thus, response times and percentage of correct answers were computed for each condition in the seated and walking trials. Response times were defined as the time between the delivery of the last piece of pertinent information and the verbal response.

### Statistical analysis

All statistical analyses were performed using IBM SPSS (Version 21 for Windows, SPSS Science, Chicago, USA) and all significance levels were set at p<.05. Moderately right skewed variables (AP and ML harmonic ratios of pelvis accelerations) were log transformed and slightly right skewed variables (response times) were square-root transformed to permit parametric analyses [26]. A three-way repeated measures analysis of variance (ANOVA) with ambulation (seated, walking), task type (VS, NS) and task difficulty (easy, difficult) as within-subject factors was initially performed on cognitive task response times and revealed a significant (three-way) ambulation by task type by task difficulty interaction (see results). Subsequently two-way repeated measures ANOVAs with task type (VS, NS) and task difficulty (easy, difficult) as within-subject factors were performed on the response times for seated and walking trials separately. Two-way repeated measures ANOVAs with task type (VS, NS) and task difficulty (easy, difficult) as within-subject factors were also performed on the gait parameters. Planned contrasts were performed where main effects or interactions were identified. Due to its markedly non-normal distribution, Friedman tests (and post-hoc Wilcoxon tests) were used to compare percentage of correct answers for the cognitive tasks between the four cognitive conditions for the walking and seated trials. Wilcoxon tests were also performed to compare percentage of correct answers between seated and walking trials for each task condition. Finally, Pearson’s correlations were conducted to assess the relationship between gait speed and gait variability and harmonic ratios within the four dual task conditions.

## Results

### Cognitive task performance

Cognitive task performance during the seated and walking trials are presented in Table 1. While seated, the percentage of correct answers differed between conditions (χ^2^ = 22.53, df = 3, *p*<.001) with participants providing significantly more correct answers in the easy compared with the difficult tasks (VS: Z = −2.876, *p* = .004; NS: Z = −2.937, *p* = .003); but no difference in the VS-easy and NS-easy tasks (Z = −1.023, *p* = .306) or the VS-diff and NS-diff tasks (Z = −0.835, p = .404). While walking, the percentage correct answers differed across conditions (χ2 = 13.775, df = 3, p = .003) with more correct answers provided in the NS tasks compared with the VS tasks (easy: Z = −2.002, p = .045; difficult: Z = −2.535, p = .011) but no difficulty level effects (p>.05). Errors made in walking and seated trials were similar (p>.05), except for the VS easy condition, where more errors were made while walking (Z = −2.756, p = .006).

**Table 1:** Descriptive data for the four cognitive tasks (nonspatial (NS) easy, NS difficult, visuospatial (VS) easy and VS difficult) in the seated and walking conditions for the 36 participants.

Parameter	NS cognitive task		VS cognitive task	
	Easy	Difficult	Easy	Difficult
***Seated trials***				
**Number of instructions per 30 s**	3.6 (0.5)	2.5 (0.5)	2.9 (0.4)	2.4 (0.6)
**Percentage of correct answers**	91 (89–100)	83 (67–100)	89 (86–100)	78 (67–89)
**Response time (s)**	4.08 (0.82)	5.89 (1.44)	4.66 (1.21)	5.21 (1.14)
***Walking trials***				
**Number of instructions per trial**	2.4 (0.2)	1.8 (0.1)	2.3 (0.0)	2.0 (0.0)
**Percentage of correct answers**	100 (83–100)	86 (75–100)	86 (67–100)	67 (50–83)
**Response time (s)**	4.97 (1.29)	5.93 (0.90)	4.52 (0.91)	5.56 (1.98)

Data are presented as mean (SD), except the percentage of correct answers are presented as median (interquartile range) due to non-normal distributions.

The three-way repeated measures ANOVA revealed a significant ambulation by task type by difficulty (three-way) interaction effect (F_1, 29_ = 7.073, p = .013) on cognitive task response time. Subsequently, two-way repeated measures ANOVAs were conducted to examine task type and difficulty effects for the seated and walking trials separately. For the seated trials, there was a significant task type by difficulty interaction effect (F_1, 31_ = 15.24, p<.001) on response time resulting from a greater differential effect of increased difficulty for the NS compared with the VS cognitive tasks. There was no main effect of task type (F_1,31_ = 0.486, p = .491) and a significant main effect of difficulty (F_1,31_ = 97.727, p<.001) indicating slower response times in the difficult versus easy cognitive tasks.

For the walking trials, there was no significant task type by difficulty interaction effect (F_1, 32_ = 0.947, p = .338) on response time, but significant main effects for both task type (F_1, 32_ = 16.30, p<.001) and difficulty (F_1, 31_ = 33.09, p<.001). This indicated that while walking, participants had slower response times in the NS compared with VS cognitive tasks and in the difficult compared with the easy cognitive tasks. Finally, response times between the seated and walking conditions did not differ significantly (F_1, 29_ = 1.408, p = .245).

### Dual task type effects: gait speed, step length and cadence

Two-way repeated measures ANOVAs showed no significant task type by difficulty interaction effects for gait speed and step length, but significant main effects for both task type and difficulty. This indicates participants walked slower and took shorter steps when concurrently performing the VS tasks compared with the NS tasks and when performing difficult cognitive tasks compared with easy cognitive tasks (Table 2). There was a significant task type by difficulty interaction effect on cadence (F_1,35_ = 5.132, *p* = .030), that resulted from a reduced cadence in the VS-diff task compared with both the NS-diff (t = −0.053, *p* = .003) and the VS-easy tasks (t = −0.051, *p*<.001).

**Table 2:** Mean (SD) values for the gait parameters in normal walking (no cognitive task) and the four cognitive dual tasks conditions: nonspatial (NS) easy, NS difficult, visuospatial (VS) easy and VS difficult for the 36 participants.

Parameter	No cognitive task	NS cognitive task		VS cognitive task		Task type effects	Task difficulty effects	Task type by difficulty interaction
		Easy	Difficult	Easy	Difficult			
15 m walk time (s)	12.38 (1.49)	15.59 (2.99)	15.96 (3.07)	16.15 (3.27)	17.27 (4.25)			
Gait speed (m.^s−1^)	1.23 (0.15)	1.00 (0.18)	0.97 (0.17)	0.96 (0.17)	0.91 (0.19)	F_1,35_ = 10.338, p = .003	F_1,35_ = 8.842, p = .005	F_1,35_ = 1.742, p = .196
Cadence (steps.s^−1^)	1.86 (0.13)	1.69 (0.21)	1.67 (0.22)	1.67 (0.23)	1.62 (0.25)	F_1,35_ = 5.783, p = .022	F_1,35_ = 9.575, p = .004	F_1,35_ = 5.132, p = .030
Step length (cm)	66.25 (6.40)	58.85 (6.62)	57.94 (6.37)	57.40 (5.65)	56.04 (6.48)	F_1,35_ = 7.429, p = .010	F_1,35_ = 6.733, p = .014	F_1,35_ = 0.416, p = .523
Step time variability (%)	3.92 (1.47)	4.85 (1.80)	5.17 (1.86)	5.34 (1.98)	5.85 (2.55)	F_1,35_ = 5.654, p = .023	F_1,35_ = 4.049, p = 0.052	F_1,35_ = 0.516, p = .477
V harmonic ratio^a^	2.70 (0.53)	2.36 (0.41)	2.32 (0.46)	2.30 (0.42)	2.11 (0.41)	F_1,34_ = 14.770, p = .001	F_1,34_ = 12.997, p = .001	F_1,34_ = 7.599, p = .009
AP harmonic ratio	2.78 (0.63)	2.43 (0.62)	2.37 (0.57)	2.37 (0.56)	2.25 (0.56)	F_1,35_ = 7.380, p = .010	F_1,35_ = 3.264, p = .079	F_1,35_ = 2.787, p = .104
ML harmonic ratio	1.77 (0.44)	1.51 (0.34)	1.48 (0.38)	1.46 (0.37)	1.40 (0.33)	F_1,35_ = 7.663, p = .009	F_1,35_ = 2.970, p = .094	F_1,35_ = 1.361, p = .251

ANOVA results examining main and interaction effects of task type and difficulty are also presented.

a n = 35; erroneously high data for one participant excluded from the analysis of this variable.

Note: There were also significant effects of added cognitive load (p<0.001) for all gait parameters, with each dual task condition (VS-easy, VS-diff, NS-easy, NS-diff) producing significantly slower gait speeds, shorter step lengths, reduced cadence, increased step time variability and reduced harmonic ratios compared with the no cognitive task walking condition (one-way repeated measures ANOVAs with planned contrasts p≤0.001).

### Dual task type effects: variability and smoothness

Two-way repeated measures ANOVAs showed no significant task type by difficulty interaction effects for step time variability and AP and ML harmonic ratios, but significant main effects for both task type and difficulty. This indicates participants had more variable gait and less smooth pelvis accelerations in the VS versus the NS cognitive tasks and the difficult versus easy cognitive tasks (Table 2). There was a significant task type by difficulty interaction effect on the V harmonic ratio (F_1,34_ = 7.599, *p* = .009), that resulted from less smooth pelvis accelerations in the VS-diff condition compared with both the NS-diff (t = −0.204, *p*<.001) and VS-easy (t = −0.189, *p*<.001) conditions.

### Associations among the gait parameters

Pearson’s correlations revealed that gait speed was associated with step time variability (r = −0.541 to −0.665, *p*≤.001) and harmonic ratios of pelvis accelerations (V: r = 0.438 to 0.687, *p*≤.008; AP: r = 0.506 to 0.590, *p*≤.002; ML: r = 0.445 to 0.670, *p*≤.007) in all four dual task conditions.

## Discussion

In this study we compared the effects of VS and NS cognitive tasks on locomotor control in older people while controlling for task difficulty. The findings build on previous work on balance control during standing and stepping [4]–[6], [8], [9], [11] by showing that VS cognitive tasks interfere with locomotor control to a greater extent than NS tasks. This differential effect was manifest in spatiotemporal parameters (reduced velocity cadence, stride length) as well as variability and smoothness measures (increased step time variability and reduced harmonic ratios).

No significant differences in either cognitive task type error rates or response times were evident in the seated conditions, suggesting equivalent task difficulty. In the walking trials, participants responded faster to the VS tasks than the NS tasks but at the cost of making significantly more cognitive task errors, i.e. they traded accuracy for speed. Thus, without specific instruction regarding which task to prioritize, healthy older people showed greater impairments in both gait and cognitive processing when performing a VS task while walking. These findings therefore support the bottleneck model of dual task interference, in that they suggest that cognitive resources required for locomotor control likely share similar pathways (i.e. the VS sketchpad of working memory [27]) to those required for performing VS tasks.

Previous studies have reported complementary findings, in that they have shown VS deficits identified in neuropsychological test batteries are associated with gait instability [16], [18], freezing of gait [19] and increased risk of multiple falls in healthy populations [16], [17] and people with Parkinson’s disease [18], [19]. They also extend knowledge that the information updating and monitoring process of executive function (working memory) is associated with gait stability (stride time variability) [28] in healthy older people, by specifying that the VS aspect of working memory is particularly relevant for locomotor control.

In each condition, slower gait speed was significantly correlated with detrimental changes in stability, consistent with a mechanical explanation that step time variability and pelvis harmonic ratios are optimised at usual speed [29]. Slower gait speed when concurrently performing the VS cognitive task could be considered a compensatory mechanism to maintain balance, as it would increase available time to respond to hazards not seen while attention is divided, but at the apparent cost of gait smoothness. Alternatively, it could be that the maintenance of optimal speed to minimize gait variability and maximise gait smoothness requires increased levels of attention for older people. As reduced walking speed, increased step timing variability [30], [31] and reduced pelvis harmonic ratios [23] during unobstructed gait are associated with an increased fall risk in older people, a gait assessment with a concomitant VS spatial task might be a useful test to include as part of a fall risk assessment.

The finding that cadence was significantly reduced in the difficult VS tasks compared with the difficult NS task is of interest given that cadence is thought to be controlled at the sub-cortical level by central pattern generators [32]. As cadence would not be expected to be affected by an increased attentional load, our findings may reflect a cortical-driven adaptation to improve walking stability or reduce speed to a further extent than that achieved by a reduction in step length.

There is neuroimaging evidence supporting the VS sketchpad working memory model [27] demonstrating that verbal and VS working memory are represented in the human brain by different domain-specific networks [33]. In addition, brain imaging studies that have examined neural correlates of either gait or spatial attention/working memory tasks in young people, point to commonalities in activated cerebral structures [34]–[38]. Brain areas activated during spatial attention and working memory tasks include the supplementary motor area, the premotor cortex, the cerebellum vermis and the precuneus [34], [36]. Other brain imaging studies [35], [38] have identified activation in these same structures (supplementary motor area, the premotor cortex, the cerebellum vermis) during gait, while conducting motor imagery of walking tasks during functional magnetic resonance imaging has identified activation of the precuneus amongst other structures [37].

It has also been documented that the hippocampus and entohirnal cortex are key cortical regions that sub-serve spatial memory and are necessary for the sequential ordering of movement [39], [40], as would be required to ensure a stable gait pattern. Atrophy in these regions is characteristic of mild cognitive impairment and Alzheimer’s Disease [41], in which patients also show early impairment in VS skills and unstable gait patterns [42]. Thus, it is possible that our VS dual task walking paradigm might have exceeded the processing capacity of the hippocampal and entohirnal regions, leading to the deteriorations in both gait and cognitive task performance.

Previous dual task balance studies [43]–[45] have found older adults tend to prioritize the motor tasks (i.e. postural control, and balance recovery on a moving platform) and perform significantly worse in cognitive tasks in dual task conditions. However, our study appears to show the opposite prioritization pattern, in that a greater dual task cost was evident for gait-performance, while cognitive task performance during the walking trials generally did not differ from the seated trials (one exception being the percentage of correct answers in VS-easy). This apparent difference in prioritization may be explained by the nature of the tasks; walking at self-selected speed along a flat corridor, free of obstacles is likely to be perceived as less threatening than maintaining balance on a moving platform.

This study has certain limitations. It is acknowledged that the VS instructions were longer that the NS instructions and that arithmetic tasks could involve some level of VS processing or share some common cortical networks [46]. Strictly, NS tasks such as forward digit span or verbal fluency might have been preferable with respect to not containing VS elements. However we wished to avoid the potential cross-talk between a motor rhythmic task (gait) and a verbal rhythmic task, which has been shown to lead to better performance in one or both tasks [47]. Secondly, it should be noted that we did not analyse the encoding periods (participants listening to the instructions) separately from the information maintenance periods and the retrieval period during which the participants responded [8], [9]. Retrieval is assumed to be more attentionally demanding than encoding [8], [9]. The participants generated more responses in the NS-easy conditions and therefore more retrieval periods could have potentially impaired walking stability. However, this was not the case as participants walked faster with a smoother and less variable gait pattern in the NS compared with the VS dual task conditions. Finally, data are presented for walking-only trials for reference purposes, and it is acknowledged that comparisons with the dual task conditions are limited by not controlling for articulatory responses.

## Conclusion

This study showed that while controlling for secondary task difficulty, VS cognitive tasks led to slower, more variable and less smooth gait patterns, compared with NS cognitive tasks. These findings support the bottleneck theory of dual task interference rather than the limited attentional resources model as they suggest that the VS processing component of working memory is involved in gait control. In the future, the use of functional neuroimaging techniques allowing recording of cortical activity during gait might provide further insight into the cognitive processes relating to walking stability. At present, the clinical implications of this research are that tasks requiring VS attention during locomotion might present an additional challenge to walking stability, particularly in older people. This finding may be pertinent to persons at increased risk of falls, such as those with sensorimotor deficits and/or neuropsychological impairments.
